# Emergency Management of Hypertension in Children

**DOI:** 10.1155/2012/420247

**Published:** 2012-04-19

**Authors:** Dinesh Singh, Olugbenga Akingbola, Ihor Yosypiv, Samir El-Dahr

**Affiliations:** ^1^Division of Pediatric Critical Care, Department of Pediatrics, Tulane University Health Sciences Center, 1430 Tulane Avenue, SL-37, New Orleans, LA 70112, USA; ^2^Division of Pediatric Nephrology, Department of Pediatrics, Tulane University Health Sciences Center, New Orleans, LA 70112, USA

## Abstract

Systemic arterial hypertension in children has traditionally been thought to be secondary in origin. Increased incidence of risk factors like obesity, sedentary life-styles, and faulty dietary habits has led to increased prevalence of the primary arterial hypertension (PAH), particularly in adolescent age children. PAH has become a global epidemic worldwide imposing huge economic constraint on health care. Sudden acute increase in systolic and diastolic blood pressure can lead to hypertensive crisis. While it generally pertains to secondary hypertension, occurrence of hypertensive crisis in PAH is however rare in children. Hypertensive crisis has been further subclassified depending on presence or absence of end-organ damage into hypertensive emergency or urgency. Both hypertensive emergencies and urgencies are known to cause significant morbidity and mortality. Increasing awareness among the physicians, targeted at investigation of the pathophysiology of hypertension and its complications, better screening methods, generation, and implementation of novel treatment modalities will impact overall outcomes. In this paper, we discuss the etiology, pathogenesis, and management of hypertensive crisis in children. An extensive database search using keywords was done to obtain the information.

## 1. Definitions and Epidemiology

Primary arterial hypertension is a global epidemic affecting predominantly adult population [1]. Although secondary etiologies of hypertension predominate in children, the prevalence of primary arterial hypertension has been increasing at an alarming rate particularly in adolescents and older children [2]. Recent survey conducted by the National Health and Nutrition Examination Survey (NHANES) in 8–17-year-old children showed a prevalence of prehypertension and hypertension of about 10% and 4%, respectively, with a higher incidence in African American and Mexican Americans [3]. Increase in the prevalence of hypertension has paralleled the increased prevalence of childhood obesity [4]. Childhood obesity has increased by more than three times in the past three decades [5]. The *Fourth Report on the Diagnosis, Evaluation, and Treatment of High Blood Pressure in Children and Adolescents* classified pediatric hypertension into various stages [6] (Table 1). In one study the incidence of stage 1 and 2 hypertension was reported to be 2.6% and 0.6%, respectively, in adolescent students [7]. The *Joint National Committee on Detection, Evaluation, and Treatment of Hypertension, JNC7*, has labeled acute severe elevation of blood pressure above 180/120 mmHg (about 20 mmHg above the Stage II hypertension) as “*Hypertensive Crisis*” in adults [8]. It is further subclassified based on presence of target organ abnormalities like seizures, intracranial hemorrhage, Posterior reversible encephalopathy syndrome (PRES), focal neurological deficit, congestive cardiac failure, papilledema, retinal hemorrhages, and acute vision loss into *Hypertensive Emergency* and in the absence of target organ abnormalities as *Hypertensive Urgency *[8]. Unfortunately, no such clear definition has been proposed for Hypertensive Crises in children. Sequelae of hypertension emergencies like left ventricular failure, encephalopathy, aortic dissection, myocardial ischemia, acute renal insufficiency, and retinal damage are well known in adults [9]. However, target organ damage and adverse effects have also been demonstrated in older children and adolescents and are an evidence of long-term blood pressure elevation [10–17]. In addition, target organ damage has been reported even in neonates and pediatric patients with prehypertension and white coat hypertension, implying that any age and any level of hypertension could result in long-term consequences. About 1% of all adults with a diagnosis of hypertension develop Hypertensive crisis, of which 76% are hypertensive urgencies and 24% are hypertensive emergencies [18]. Similar data in children is not available. In addition, although the prevalence of the primary hypertension has increased over the past three decades, the incidence of hypertensive crisis is very uncommon in pediatric patients with primary hypertension and its occurrence is more common in pediatric patients with secondary hypertension. Terminologies like *Accelerated Hypertension* and *Malignant Hypertension* are obsolete and should not be used.

**Etiology**

In adults, majority of the cases of hypertensive crises are due to nonadherence to prescribed medication, drug overdose, sudden withdrawal of antihypertensive medications, and so forth [19–22]. In comparison, majority of pediatric hypertensive crises are renal in origin [23]. Interestingly, etiologies also differ according to the patient's age, onset (acute versus chronic), and duration (intermittent/episodic or persistent). For example, conditions like coarctation of aorta, renal vein, or artery thrombosis predominate in neonates. However, renal parenchymal diseases, pregnancy, endocrine conditions, autoimmune diseases, medications, drugs, and alcohol are important etiologies in older children and adolescents. Conditions like pheochromocytoma can present with episodic or sustained hypertension whereas chronic glomerulonephritis has persistent/sustained hypertension (Table 2).

**Pathophysiology and Pathogenesis**

**Figure 1:**
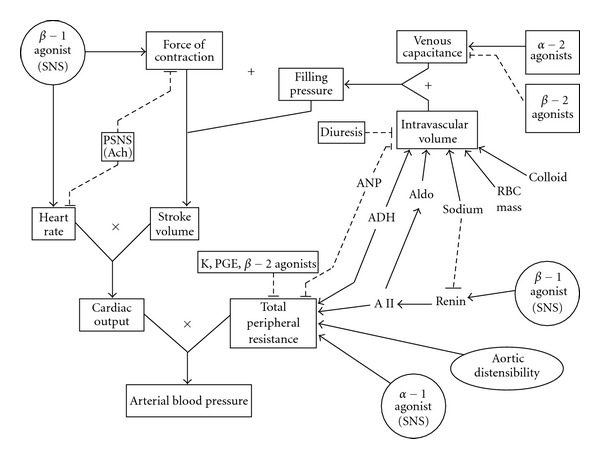
Factors that determine the arterial blood pressure (adapted with permission from [25]).

Blood pressure is a product of cardiac output and peripheral vascular resistance (PVR). Cardiac output is a product of heart rate and stroke volume. In turn, stroke volume is determined by preload, contractility, and after-load/PVR [24].  Figure 1 outlines the various factors that determine the arterial blood pressure (adapted with permission from [25]).Any factor which enhances the heart rate and determinants of stroke volume would result in hypertension. The pathogenesis of hypertensive crisis is multifactorial and much of the supportive data is based on both animal and human adult studies. The factors that have been implicated in the pathogenesis include elevated blood pressure, fluid overload, sympathetic overactivity, renin-angiotensin-aldosterone system activation, oxidative stress, endothelial dysfunction, and inflammation. There is a complex interaction between all these factors and all or some factors occurring simultaneously may be involved in the pathogenesis of hypertensive crisis.

## 2. Elevated Blood Pressure

Complex interactions between renal, humoral, neural, and cardiovascular systems are involved in the maintenance of normal perfusion pressures to target organs during fluctuations of blood pressures [24]. Any disturbance in the autoregulatory mechanisms results in mechanical stress resulting in vascular injury and endothelial damage. Endothelial damage thus initiates a cascade of proproliferative, prothrombotic, and proinflammatory reactions, in addition to release of vasoactive peptides. These cascades of events eventually result in fibrinoid necrosis and tissue ischemia. This culminates into a vicious cycle of tissue ischemia potentiating the blood pressure due to release of vasoactive peptides, sympathetic overactivity, and fluid retention leading to further endothelial damage and inflammation which in turn again worsen the tissue ischemia [26, 27].

## 3. Renin-Angiotensin-Aldosterone System (RAAS)

RAAS plays an important role in the regulation of blood pressure and during hypertensive crisis [28]. The enzyme renin acts on angiotensinogen (AGT) to generate angiotensin I (Ang I). Ang I is further converted to angiotensin II (Ang II) by angiotensin converting enzyme (ACE). Ang II exerts its effects by binding to two major types of receptors—AT_1_R and AT_2_R [29]. Functions like vasoconstriction, cellular proliferation, cellular hypertrophy, fibrosis, atherosclerosis, antinatriuresis, and release of aldosterone, endothelin, norepinephrine, and vasopressin are initiated by binding of Ang II to AT_1_R. In addition, Ang II induces mitochondrial dysfunction via a protein kinase C-dependent pathway by activating the endothelial cell NADPH oxidase and formation of peroxynitrite [30]. A recent study demonstrated that Ang II stimulates increased IL-6 production both in vivo and in vitro. In addition to its role in hypertension, increased IL-6 may play an important pathogenic role in CKD by Ang II-mediated induction of multiple fibrotic genes and ET-1 production leading to renal injury and fibrosis [31]. In addition, recent identification (Pro) Renin receptors and functionally active Ang II-derived peptides like Ang 1–7 have been shown to play pathological role in causing hypertension [32]. 

## 4. Inflammation

Various inflammatory cytokines and chemokines have been implicated in the pathogenesis of hypertension. Ang II has been shown to be proinflammatory and profibrotic by inducing and activating various inflammatory pathways and upregulating cytokines, chemokines, and NFKB. The pivotal role of various T-cell subsets and macrophages in the regulation of blood pressure and target organ damage has been demonstrated in recent studies [33–38]. Amelioration of the target organ damage by immune-suppressant treatment further confirms involvement of immune mediators in the pathogenesis of hypertension. Ang II also facilitates recruitment of leukocytes through the endothelium by induction of ICAM-1 and VCAM-1 [39, 40]. T lymphocytes CD4 (+) and, to a lesser extent, CD8 (+) have been demonstrated to mediate the accelerated microvascular thrombosis associated with Ang II-induced hypertension [41]. Although the exact mechanism is unclear, it is thought to be secondary to complex interaction between platelets and cytokines resulting in activation of coagulation cascade. In addition, the presence of NADPH oxidase-derived reactive oxygen species also facilitates prothrombotic action of Ang II [41]. Recent study also demonstrated immunosuppressive properties of regulatory T cells (Treg) when adaptive transfer of isolated Treg cells into Ang II–infused mice resulted in amelioration of cardiac damage [42].

## 5. Oxidative Stress and Endothelial Dysfunction

 Nitric oxide (NO) is synthesized by endothelial nitric oxide synthase (eNOS) in the vascular endothelium from its precursor molecule L-arginine. NO in the presence of soluble guanylate cyclase (sGC) results in increased intracellular levels of cyclic Guanosine monophosphate (cGMP). Elevated cGMP causes decrease in intracellular calcium ion levels leading to decreased vascular tone. Oxidative stress may play a causal role in the development of hypertension by altering the vascular tone either by oxidative modification of proteins and nucleic acids or by decreasing the bioavailability of nitric oxide. Superoxide anions generated by various enzymes such as NADPH oxidase, xanthine oxidase, and enzymes involved in mitochondrial respiratory chain may directly inactivate NO and inhibit sGC. Increased angiotensin II levels facilitate further generation of superoxide anions by stimulating these enzymes. In addition, superoxide anions lead to uncoupling of eNOS by oxidating the BH4 (tetrahydrobiopterin). BH4 is an essential cofactor necessary in generation of NO by eNOS enzyme. Uncoupling of eNOS also facilitates further generation of superoxide anions. These superoxide anions cause increased vascular cell proliferation and migration, apoptosis, inflammation, extracellular matrix alterations, and endothelial dysfunction [43–46].

## 6. Central Nervous System

The role of central nervous system in the regulation of blood pressure via modulation of sympathetic and parasympathetic nervous system is well known. However, recent studies suggest that increased sodium intake results in increase in endogenous ouabain (EO) levels in the paraventricular and supraoptic nuclei and at the circumventricular organs such as subfornical organ. This induces an acute but transient Ang-II- mediated increase in peripheral sympathetic nervous system resulting in elevation in blood pressures. Experiments conducted in rats reveal complex interactions involving sodium ions, epithelial sodium channels (ENaCs), RAAS, and EO in the brain which activate sympathetic nervous system. Thus, a brain Na^+^-ENaC-RAAS-EO pathway and a neuromodulatory pathway involving Aldosterone-EO-Ang II have been proposed in explaining the mechanism of action of hypertension. Further research and understanding of these novel mechanisms will help in newer antihypertensive therapies [47].

**Figure 2:**
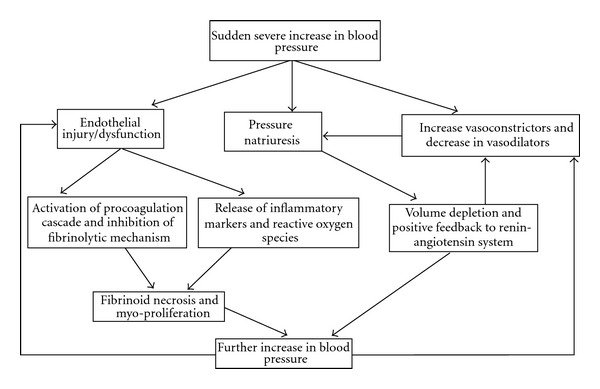
Mechanism of hypertensive crisis.

In addition, genetic mutations and polymorphisms [48], and insulin resistance [49], and abnormalities involving the sodium transport mechanisms like Na^+^/H^+^ exchanger, Na^+^/K^+^/2Cl^−^cotransporter, Na^+^Cl^−^ cotransporter, Na^+^/K^+^ATPase, and sodium-phosphate cotransporter [50, 51] have also been implicated in the pathogenesis of hypertension. A possible mechanism of hypertensive crisis is shown in Figure 2.

### 6.1. Clinical Features and Target-Organ Damage

Clinical presentation varies depending upon age, the target organ involved, and etiology. Neonates may present with apnea, cyanosis, irritability, and poor feeding [52].In addition, clinical features may reflect specific etiologies like endocrine diseases, autoimmune conditions, pregnancy, and drug abuse. Older children with long-term hypertension or acute exacerbation of chronic hypertension or sudden severe elevation of blood pressure may present with symptoms related to end organ abnormalities involving the heart, eye, kidney, and brain [53].

### 6.2. Cardiovascular Manifestations

 Depending on the duration and acuity of the symptoms, the cardiac involvement can be in the form of left ventricular hypertrophy (LVH), left ventricular failure, or left ventricular ischemia [54, 55]. Although left ventricular hypertrophy has traditionally been defined in pediatric population as left ventricular mass index (LVMI) greater than 38.6 g/m^2.7^ and has been recommended in the fourth report, a recent study has demonstrated that LVMI varies significantly in children particularly those <9 years of age. The study which was performed in 2,273 nonobese, healthy children demonstrated that in children aged >9 years the 50th percentile values of LVMI ranged from 27 g/m^2.7^(girls) to 32 g/m^2.7^(boys) and varied little with age. The 95th percentile values of LVMI in the >9 years age group ranged from 40 g/m^2.7^(girls) to 45 g/m^2.7^(boys). The authors concluded that values >40 g/m^2.7^in girls and >45 g/m^2.7^ in boys should be considered abnormal. In contrast, the 50th percentile values of LVMI in children <9 years age group varied significantly from 56.44 g/m^2.7^(boys) and 55.38 g/m^2.7^(girls) in infants less than 6 months of age to 31.79 g/m^2.7^(boys) and 29.71 g/m^2.7^(girls) in children less than 8years of age. Similarly, the 95th percentile values of LVMI in the <9 years age group varied from 80.1 g/m^2.7^(boys) and 85.6 g/m^2.7^(girls) in infants less than 6 months of age to 44.6 g/m^2.7^(boys) and 43.5 g/m^2.7^(girls) in children less than 8 years of age (Table 3). This study provides normal percentile values for young children and emphasizes the need for age–appropriate LVMI cut points and use of appropriate percentile curves particularly in children <9 years of age [56]. The LVH is common in children with hypertension with an incidence of 41.1% particularly in children with high Body-Mass index (BMI) and in Hispanic population [57]. Left ventricular failure can lead to symptoms such as increased work of breathing, shortness of breath, chest pain, palpitations, decreased urine output, and poor appetite. Sudden acute increase in blood pressure may precipitate left ventricular failure in any pediatric age group but is more commonly reported in neonates [58–60]. Carotid intima media thickness (CIMT), measured by B-mode ultrasound at end diastole, has emerged as a surrogate marker of early atherosclerotic changes and is predictive of adult cardiovascular structural damage. Indeed, increasing number of studies in children with hypertension, dyslipidemia, diabetes, and obesity have shown an increased CIMT. However, a recent study showed that nonobese children with primary hypertension had increased CIMT compared with BMI-matched controls. Although obesity may play a significant role in vascular changes, this study provides strong and interesting evidence that CIMT may be increased in nonobese children with primary hypertension. The major drawback of this surrogate marker is that the reference values are not available for children younger than 10 years and further studies are needed to determine reference values of CIMT in this age group [61–63]. 

### 6.3. Neurological Manifestations

Loss of cerebral autoregulation leading to disruption of the blood brain barrier and endothelial dysfunction results in imbalance in oxygen delivery, edema formation, and microhemorrhages [64]. These changes may lead to seizures, altered mental status, PRES, vomiting, signs of raised intracranial pressure, focal neurological deficits, and headache. Headache is the most common symptom [65–69]. In one study seizures occurred in 25% of children, encephalopathy in 25%, facial palsy in 12%, and hemiplegia in 8% [18]. Posterior reversible encephalopathy syndrome has been reported to predominantly affect the occipitoparietal white matter with occasional spread to basal ganglia, cerebellum, and brainstem. Various etiologies like post-chemotherapy, posttransplant, postinfectious, autoimmune conditions, and posthypertensive crisis have been known to cause PRES. Clinical features include headache, altered mental status, nausea, vomiting, seizures, cortical blindness, and focal neurological deficits. Magnetic Resonance Imaging shows bilateral, symmetrical, involvement of white matter in occipitoparietal regions which appear as hyperintense lesions on T2-weighted images and hypointense or isointense lesions on Diffusion-Weighted Images. PRES is a completely reversible condition with occasional reports of neurological deficits [70–72].

### 6.4. Renal Manifestations

Hematuria, flank pain, and oliguria would indicate renal involvement. The most common etiology leading to hypertension in children is renal disease but hypertension itself can result in renal injury and failure secondary to loss of autoregulation of renal blood flow. But the data exploring the impact of hypertension on the renal function and structural injury in pediatric population is limited. Histologically fibrinoid necrosis with thrombosis involving the intrarenal arteries has been demonstrated in adult studies which result in clinical presentation consistent with microangiopathic hemolytic anemia [73, 74]. A recent study, however, has demonstrated increased microalbuminuria and decreased glomerular filteration rate in prehypertensive children particularly with high blood pressure load [75]. In addition, another study demonstrated a reduction in microalbuminuria and LVH when hypertension was controlled with ACE inhibitors [76]. These findings suggest that renal dysfunction and structural injury may occur early even in pediatric population with hypertension and continue into adult life and studies are needed to further elucidate these findings.

### 6.5. Ophthalmological Manifestations

Retinal bleeds, papilledema, loss of visual acuity, acute ischemic optic neuropathy, and cortical blindness have been reported secondary to hypertensive crisis [77]. Loss of vision can be serious and permanent. Traditionally hypertensive retinopathy assessed by direct fundoscopy has been described based on Keith-Wagner-Barkar's classification (1939) which was subsequently modified by Scheie (1953) [78, 79]. The major drawback of these classifications is that direct fundoscopic examination is limited by physician's experience and high inter- and intraobserver variability. More recently Wong and Mitchell (2004) (Table 4) proposed a new classification which stratified cardiovascular risks associated with different grades of hypertensive retinopathy in adults [80]. The data regarding the prevalence of hypertensive retinopathy in general pediatric population is largely unknown. However, in two studies the prevalence of hypertensive retinopathy in children with hypertension varied from 8.9% (assessed by direct fundoscopy) to 50% (assessed by retinal photographs) [13, 81]. Majority of the children in both studies had mild retinopathy and none had higher-grade retinopathy. In addition, evidence of grade III and grade IV hypertensive retinopathy was lacking in 32% of adults with hypertensive encephalopathy [82]. Although many studies involving the risk stratification and prognostic importance based on the retinopathy grades are available in adults, no such data exists in pediatric population. In general, moderate-to-severe grades of retinopathy are relatively rare in children and further studies are needed to elucidate the importance of mild retinopathy and long-term prognosis. But newer techniques like digital imaging and computer analysis of the early retinal changes and newer grading systems will further help in risk stratification and disease progression [83].

## 7. Clinical Assessment

Clinical assessment begins with obtaining relevant present, past medical history. Potential risk factors include history of low birth weight, intrauterine growth retardation, prematurity, oligo or polyhydramnios, umbilical artery catheterization, recurrent urinary tract infections, weak stream of the urine in male child, hematuria, flank pain, polyuria, failure to thrive, joint pains, skin rashes, headaches, visual disturbances, chest pain, palpitations, and poly or oliguria. Family history of diabetes, hypertension, obesity, hypercholesteremia, early strokes, coronary artery diseases, sudden cardiac deaths, malignancies, autoimmune conditions, or hereditary conditions involving the kidneys, liver, and brain should be assessed. A detailed endocrine-related history should also be obtained. Medication history involving steroids, antihypertensives, tacrolimus, cyclosporine, oral contraceptives, and dietary and life-style history regarding smoking, alcohol, and drug abuse should be elucidated. In addition in teenage adolescent girls, pregnancy-related symptoms should be elicited. In obese children, history of sleep apnea and daytime somnolence should be obtained.

Physical examination should involve general examination to look for edema, skin rashes, neurocutaneous markers, cyanosis, elfin facies, webbing of neck, hirsutism, cushingoid features, thyroid enlargement, proptosis, and so forth. Heart rate, respiratory rate, and four-extremity blood pressure preferably both in lying and sitting position, peripheral pulses, height, weight, and BMI should be recorded. A detailed cardiovascular, respiratory, abdominal, and neurological examination should be performed to look for any evidence of coarctation, LVH, pulmonary edema, pleural, and pericardial effusions. In addition, examine for hepatosplenomegaly, intra-abdominal masses, ascites, and genitourinary abnormalities. Look for any evidence of spina bifida, hydrocephalus, signs of raised ICP, papilledema, focal neurological deficits, and cranial nerve palsies particularly 3rd and 7th cranial nerves.

## 8. Laboratory Workup and Evaluation

Successful management of the elevated blood pressure in children depends on the accurate diagnosis and evaluation of the etiology of hypertension. It is well known that the clinical spectrum of the etiology of hypertension is varied depending on the age of the child. Thus, it is vitally important that after a thorough history and physical examination, an extensive laboratory workup should be undertaken particularly in a young child who has extremely high pressure to rule out secondary causes of hypertension. In addition, workup should include tests and imaging studies to rule out end-organ damage. Some of the common initial workup and advanced investigatory studies based on the etiology have been outlined (Tables 5 and 6).

## 9. Treatment

**Figure 3:**
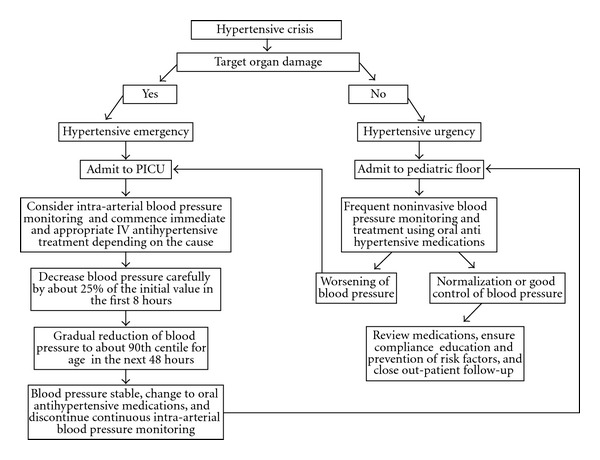
Proposed algorithm for the management of hypertensive crisis in children.

Prompt recognition and treatment is of utmost importance to prevent target organ damage. Hypertensive emergency in children or adults is an indication for admission to intensive care unit for close monitoring and prompt initiation of appropriate intravenous antihypertensive therapy depending upon the etiology. Blood pressure should be preferably monitored continuously by invasive intra-arterial line or intermittently by noninvasive methods if intra-arterial line cannot be obtained for any reason. Patient's cardiac, respiratory, and neurological status should be constantly monitored and prompt interventions implemented in case of any deterioration. Hypertensive urgency may be managed on regular pediatric unit and with oral antihypertensive medications. Moving the patient to intensive care unit may be considered in case of worsening of clinical condition (Figure 3). As per the *Fourth Report on the Diagnosis, Evaluation, and Treatment of High Blood Pressure in Children and Adolescent*, the primary aim of antihypertensive treatment is to reduce the blood pressure to <95th percentile and to <90th percentile in the presence of comorbid conditions like diabetes, cardiac, or renal disease. Furthermore, the mean arterial blood pressure should be lowered no more than ≤25% of the initial value in the first 1 hr and a gradual reduction should be obtained over the next 24–48 hrs to normalize the blood pressure [6]. However, no clear guidelines exist about the rate of lowering the blood pressure in the presence of ischemic stroke.

Majority of the randomized clinical trials and data on anti-hypertensive medications are obtained from adult studies. Data regarding the safety, efficacy, and adverse events about most of the anti-hypertensive agents is not available in pediatric population and is either extrapolated from adult clinical trials or has been limited to expert opinions. Some of the intravenous antihypertensive medications used in the treatment of hypertensive emergencies include sodium nitroprusside, nicardipine, esmolol, hydralazine, labetalol, fenoldopam, and phentolamine. Some of the medications used in the treatment of hypertensive urgencies include enalapril, nifedipine, clonidine, minoxidil, and angiotensin II receptor blockers. In our institute depending upon the etiology and contraindications, we prefer to use sodium nitroprusside, nicardipine, and esmolol as our first, second, and third choices in the management of hypertensive emergency. In case of hypertensive urgencies, we prefer to use amlodipine, nifedipine, ACE inhibitors, angiotensin II receptor blockers, labetalol, or clonidine either alone or in combination. The details of the mechanisms of action and adverse effects are outlined in Table 7. However, the results of a recent Cochrane review showed that there is no evidence demonstrating that antihypertensive drugs reduce mortality or morbidity in patients with hypertensive emergencies. The authors were unable to determine which drug or drug class is most effective in reducing mortality and morbidity. The authors concluded that RCTs are needed to assess different drug classes to determine initial and longer-term mortality and morbidity outcomes [84]. In the next section, we have summarized the management of hypertension crisis in some of the important pediatric conditions which we think may be more useful to the clinicians.

### 9.1. Hypertensive Crisis due to Medications or Drugs

Sudden cessation of opiates, benzodiazepines, and clonidine can also lead to withdrawal syndrome and present with hypertensive crisis. Reintroduction of medications causing the withdrawal followed by gradual weaning of the medications will result in resolution of hypertensive crisis in most cases. Cocaine toxicity is a well-known cause of hypertensive crisis particularly among adolescents. Mechanism of hypertensive crisis due to cocaine toxicity involves potentiation of catecholamine effects by inhibition of the presynaptic uptake of norepinephrine. Intravenous alpha-blocker like phentolamine is treatment of choice and beta-blockers may be added if needed later in the treatment [85–87]. Hypertensive crisis is also known to occur when medications like monoamine oxidase inhibitors interact with food containing tyramine and other medications like dextromethorphan, methylene blue, selective serotonin reuptake inhibitors, and linezolid. Hypertension due to MAOI interaction can be treated with either an alpha blocker or sodium nitroprusside [88–91]. Hypertensive crisis due to amphetamine toxicity is due to sympathomimetic and serotonergic effects of amphetamines. In addition to decontamination, cooling, sedation, intravenous alpha-blockers, or sodium nitroprusside is the treatment of choice [92, 93]. Beta-blockers alone are absolutely contraindicated in all these toxidromes as they will worsen the hypertensive crisis due to unopposed action on alpha receptors.

### 9.2. Hypertensive Crisis due to Pheochromocytoma

Pheochromocytoma is rare tumor arising from chromaffin cells in the adrenal medulla and extra adrenal paraganglionic tissue in children. About 80% of the pheochromocytomas arise from adrenal medulla in children. Approximately 40% of pheochromocytomas are associated with genetic mutations and about 19–38% are bilateral. About 1% of pediatric hypertensive patients have pheochromocytoma with a peak incidence at around 11 years. But it is more commonly diagnosed in adults with a peak incidence between 4th and 6th decade of life. Multiple endocrine neoplasia type 2, Von Hippel-Lindau syndrome, neurofibromatosis type 1, and germline mutations of succinate dehydrogenase (SDH) gene are some of the common causes of hereditary pheochromocytomas. Clinical presentation of pediatric pheochromocytoma is varied and ranges from asymptomatic to sustained hypertension in about 60–90%. Paroxysmal hypertension is less common when compared to adults and its presence should raise a suspicion of pheochromocytoma. Children with dopamine secreting pheochromocytomas are usually normotensive. Symptoms such as headache, palpitations, sweating, vomiting, pallor, weight loss, polyuria, visual disturbances, anxiety, and attention deficit-hyperactivity are common presenting features in children. Quantification of plasma-free metanephrine and normetanephrine based on age-specific reference intervals and 24 hr urinary-fractionated metanephrines preferably in supine position are most sensitive diagnostic tests for pheochromocytoma. After the biochemical confirmation of the diagnosis, tumor localization studies like magnetic resonance imaging (MRI), ^123^metaiodobenzylguanidine (MIBG) scintigraphy, and occasionally in rare cases studies like [111In]-Octreotide scintigraphy, [18F]-fluorodopamine, or [18F]-fluorodeoxyglucose positron emission tomography (FDG-PET) may be required. Surgery is the treatment of choice but good preoperative medical management of hypertension for at least 10–14 days is recommended. Adequate perioperative management of hypertension will result in decrease in postoperative complications. *α* ^2^-blockade with noncompetitive *α* ^2^-blocker like phenoxybenzamine is the medication of choice. For localized tumors laparoscopic cortical-sparing adrenalectomy is the preferred surgery in children. Treatment options for invasive and metastatic tumors include embolization, systemic therapy with ^131^MIBG, or chemotherapy [94–97].

### 9.3. Hypertensive Crisis due to Renovascular Disease

Renovascular hypertension is an uncommon but important cause of correctable hypertension in children. 3–10% of the children evaluated for hypertension are found to have renovascular hypertension. Unlike atherosclerosis which is the most common cause of renovascular hypertension in adults, the most common etiology of the renovascular disease in children in Western countries is fibromuscular dysplasia. On the contrary, in developing Asian countries and South Africa, Takayasu's arteritis is the most common cause and is probably related to high incidence of tuberculosis in these countries. The other important etiologies of renovascular disease include syndromes like William's syndrome, Marfan's syndrome, Neurofibromatosis type 1, rubella syndrome, tuberous sclerosis, Klippel Trenaunay Weber syndrome, Linear sebaceous nevus syndrome and vasculitides like Kawasaki's disease, and Polyarteritis nodosa. In addition, extrinsic compression due to neuroblastoma, Wilms' tumor, and other tumors may lead to renal artery stenosis (RAS). Miscellaneous causes of RAS include irradiation, Crohn's disease, postrenal transplant, trauma and umbilical artery catherization. Children with the aforementioned conditions and hypertension should be meticulously evaluated for RAS. In addition, children with severe hypertension which is unresponsive or uncontrolled with multiple antihypertension medications or have renal arterial bruit, absent pulses, hypokalemia, elevated plasma renin activity, and unequal sizes of kidneys on ultrasound should also be investigated for RAS. RAS can be unilateral or bilateral, extrarenal, or intrarenal or both. Incidence of bilateral RAS is higher than unilateral RAS. It is important to know that renal artery bruit is an infrequent finding and may be absent in as many as 70% of the patients with RAS. Although renal arteriography is the gold standard in the investigation of RAS, other less invasive methods like Doppler ultrasonography, magnetic resonance angiography, computerized tomographic angiography, captopril renography, and digital substraction angiography may be used initially. CO_2_ angiography has been advocated in patients with renal insufficiency to minimize contrast-induced renal injury but pediatric experience is limited. When the index of suspicion is high or when noninvasive methods have been tried, invasive techniques like renal arteriography and renal vein renin assays can be obtained to confirm the diagnosis. In the presence of unilateral disease a renal vein renin ratio of >1.5 between the affected and nonaffected kidney is considered significant. In absence of any difference in the ratios bilateral RAS may be suspected. Treatment depends upon the etiology, severity, and extent of the disease. In cases of RAS secondary to Takayasu's arteritis steroids, immunosuppressive therapy and antituberculous treatment are the mainstay. In general it is advisable to avoid diuretic therapy as it may increase plasma renin levels secondary to volume depletion. In children who have RAS which is amenable to surgical treatment, percutaneous transluminal renal angioplasty, stenting, segmental ethanol ablation, revascularization, and partial or total nephrectomy of the affected kidney are some of the options. In selected cases with diffuse and extensive bilateral intrarenal arterial lesions where surgical correction cannot be performed, a trial of angiotensin converting enzyme inhibitor or angiotensin receptor blocker has been advocated under close supervision by an experienced specialist in a tertiary hospital [98, 99].

### 9.4. Hypertension in Chronic Kidney Disease (CKD)

Hypertension is a common occurrence in CKD increasing in severity with worsening in renal function. Both renoparenchymal diseases and tubulointerstitial diseases lead to renal scarring and reduction in nephron mass which results in activation of RAAS. The activation of RAAS leads to sodium and fluid retention and sympathetic hyperactivation contributing to hypertension. Volume overload is the most important factor leading to hypertension and hypertension-related morbidity and mortality in patients with CKD. In addition to the previous mechanism, several other factors like uremic toxins, increased circulating endogenous NOS inhibitors like asymmetric dimethylarginine, secondary hyperparathyroidism leading to hypercalcemia, and increased expression of endothelin A receptors have also been implicated in the pathogenesis of hypertension in CKD. Maintaining dry weight by strict volume control, dietary salt restriction, and frequent dialysis will markedly reduce the need for antihypertensive therapy. RAAS antagonist like ACE inhibitors and ARB are considered as first choice in the treatment of hypertension in this group of patients. RAAS antagonists not only lower transglomerular pressure and proteinuria but also suppress cytokines and chemokine release. These effects confer nephroprotection by reduction in glomerular hypertrophy and sclerosis and tubulointerstitial inflammation and fibrosis. In addition to RAAS antagonist, calcium channel blocker and beta-blockers are other therapeutic options [100, 101].

### 9.5. Hypertension in Microangiopathies

Thrombotic microangiopathy (TMA) is characterized by Coomb's negative microangiopathic hemolytic anemia (MAHA), thrombocytopenia, and elevated lactate dehydrogenase levels. Histopathologically the lesion is characterized by endothelial injury and complete or partial obliteration of the arteriolar lumen by fibrin and platelet clots. Three types of TMA have been described—Glomerular TMA, Acute cortical necrosis and Arterial TMA—and have prognostic value. Acute cortical necrosis type of TMA is irreversible, glomerular TMA is partially reversible, and arterial TMA is predominantly seen in adults, is associated with recurrence or relapse and severe hypertension, and portends poor prognosis. Similar histopathological lesions have been described in conditions like Hemolytic-Uremic syndrome (HUS), Thrombotic Thrombocytopenic Purpura (TTP), and Hypertensive crisis of varied etiologies. Clinical features of these conditions can overlap and may be difficult to distinguish one from another. In general, thrombocytopenia is more severe in TMA induced by HUS/TTP compared to TMA secondary to hypertensive crisis. Von Willebrand factor cleaving protease enzyme (ADAMTS 13) deficiency is frequently associated with TTP or atypical HUS and normal in typical HUS. Renal involvement is common in all these conditions. Hypertensive crisis can be the end result of renal disease or can result in acute renal failure. HUS is one of the most common causes of acute renal failure in childhood and is characterized by a triad of Coomb's negative MAHA, thrombocytopenia, and acute renal failure. It has been classified as typical or D+ HUS (diarrhea associated) or atypical or D*‒*HUS (nondiarrhea associated). D+ HUS is more common, frequently secondary to infectious etiology like Shiga-toxin-producing enterohemorrhagic *Escherichia coli*, *Shigella dysenteriae* type 1, and *Streptococcus pneumoniae*, accounts for 90% of all cases, and carries more favorable prognosis when compared to the atypical HUS. Atypical HUS accounts for the remaining 10% of cases and is secondary to genetic mutations in the proteins involved in the regulation of alternative complement pathway, or deficiency of Von Willebrand factor cleaving protease enzyme (ADAMTS 13), and vitamin B12 metabolic defects. Renal failure is common in both typical and atypical HUS but the severity of the disease and hypertension is more pronounced in atypical HUS. Management of hypertension in these conditions includes avoidance of volume overload and maintenance of normal volume status, peritoneal dialysis or continuous renal replacement therapy, and etiology-specific therapies. In addition, in children with atypical HUS and TTP early plasmapheresis and plasma exchange has been used with variable results. Prolonged therapy with Eculizumab, a monoclonal antibody against C5 preventing the formation of membrane attack complex has been used in some atypical HUS cases. In patients with HUS secondary to vitamin B12 metabolic defects hydroxocobalamin therapy has been advocated [102–105].

### 9.6. Hypertension in Kidney Transplant Recipients

Hypertension is common in pediatric patients with renal transplant and occurs in up to 90% of the recipients. Hypertension in postrenal transplant recipients increases the risks of graft dysfunction and also cardiovascular morbidity and mortality. Some of the important factors responsible for the hypertension after renal transplant include preexisting hypertension, native kidney disease, medications like steroids, calcineurin inhibitors, both cold and warm ischemia times, graft dysfunction, renal artery stenosis, thrombotic microangiopathy, and postbiopsy arteriovenous fistula, The pathogenesis of the hypertension includes sodium and water retention, activation of RAAS, sympathetic overactivity, inhibition of atrial natriuretic peptide, imbalance in the synthesis and degradation of Nitric Oxide, endothelial dysfunction, and oxidative stress. In general, the hypertension induced by cyclosporine is more severe when compared to tacrolimus. Management of hypertension in this group of patients includes close monitoring of the drug levels of immunesuppressive medications like cyclosporine and tacrolimus and using least amount of medication or switching to another medication (like cyclosporine to tacrolimus or sirolimus) needed for graft survival. Monitoring for drug interactions and avoiding or minimizing medications can increase the serum levels of the immune-suppressive medications. Calcium-channel blockers decrease the vasoconstriction induced by the calcineurin inhibitors and are considered in the management of hypertension in these patients. However, care should be taken in monitoring the levels of cyclosporine and tacrolimus as some of the CCB like diltiazem, verapamil, and nicardipine are known to interfere with the drug metabolism. In addition, thiazide and loop diuretics, ACE inhibitors, and ARB are also used either alone or in combination with control resistant hypertension. ACE inhibitors and ARB because of their antiproteinuric and antierythrocytosis effect are particularly useful. However, they can cause decrease in GFR and cause anemia and potentiate hyperkalemia. These medications should be avoided in patients with decreased GFR secondary to graft dysfunction and also in patients with posttransplant renal artery stenosis. Minoxidil may be considered in patients with tacrolimus-induced alopecia [106–108].

## 10. Conclusions

Hypertensive crisis is an important pediatric emergency associated with significant morbidity and mortality. Early diagnosis, aggressive but careful management depending on specific etiologies, and long-term follow-up will help in decreasing some of this burden. Both basic and clinical research efforts to further delineate risk factors, pathophysiology, and epidemiology should be prioritized. Thus improved knowledge will contribute to better therapies. Dietary and lifestyle modifications do not seem as important as the implementation of guidelines and policies in changing the prognosis of hypertensive crisis.

## Figures and Tables

**Table 1:** Definitions of normal and elevated blood pressure in children.

Normal blood pressure	Systolic and diastolic blood pressure below 90th centile
Prehypertension	Systolic or diastolic blood pressure above the 90th centile (or 120/80 mmHg), but below the 95th centile
Stage I hypertension	Systolic or diastolic blood pressure higher than or equal to the 95th centile, but lower than the 99th centile plus 5 mm Hg
Stage II hypertension	Systolic or diastolic BP higher than or equal to the 99th centile plus 5 mm Hg

**Table 2:** Causes of hypertension in children.

Renal	Congenital dysplastic kidneys
	Multicystic kidney disease
	Polycystic kidney disease
	Hydronephrosis
	Renal artery stenosis
	Renal vein thrombosis
	Glomerulonephritis
	Acute tubular necrosis Hemolytic-Uremic syndrome
	Obstructive uropathy
	Wilms tumor
	Diabetic nephropathy
	Pyelonephritis

Cardiovascular	Coarctation of aorta
	Takayasu's arteritis
	Moyamoya disease

Endocrine	Cushing's syndrome
	Hyperthyroidism
	Hyperparathyroidism
	Congenital adrenal hyperplasia
	Pheochromocytoma

Medications, drugs, and toxins/poisons	Corticosteroids
	Tacrolimus
	Cyclosporine
	Erythropoietin
	Amphetamines
	Oral contraceptives
	Anabolic steroids
	Phencyclidine
	Vitamin D intoxication
	Cocaine
	Alcohol
	Smoking
	Lead, thallium, mercury toxicity

Central nervous system	Brain tumors
	Intracranial hemorrhage
	Raised ICP
	Autonomic dysfunction
	Neuroblastoma
	Encephalitis

Autoimmune	Systemic lupus erythematosus
	Polyarteritis nodosa
	Rheumatoid arthritis
	Goodpasture's disease
	Wegener's Disease
	Mixed connective tissue disorders

Miscellaneous	Obesity
	Pregnancy
	IUGR
	Umbilical artery catheterization
	Hypercalcemia
	Hypervolemia
	Pain
	Drug withdrawal (opiates, clonidine, beta-blocker)

Genetic	Gordon syndrome
	Liddle's syndrome
	Turner's syndrome
	William's Syndrome
	Friedreich's ataxia
	Von Hippel-Lindau syndrome
	Tuberous sclerosis complex
	Neurofibromatosis
	Multiple endocrine neoplasia

**Table 3:** Age-specific reference values of the LVMI in boys and girls Adapted from [56].

Age	Sex	LVMI (50th percentile)	LVMI (95th percentile)
>9 years	Boys	32.0 g/m^2.7^	45.0 g/m^2.7^
	Girls	27.0 g/m^2.7^	40.0 g/m^2.7^

<8 years	Boys	31.79 g/m^2.7^	44.6 g/m^2.7^
	Girls	29.71 g/m^2.7^	43.5 g/m^2.7^

<6 months	Boys	56.44 g/m^2.7^	80.1 g/m^2.7^
	Girls	55.38 g/m^2.7^	85.6 g/m^2.7^

**Table 4:** Wong and Mitchell's classification (adapted from [80]).

Grading	Retinal signs	Sytemic associations*
Mild retinopathy	Generalized arteriolar narrowing, focal arteriolar narrowing, arteriovenous nicking, opacity (copper wiring) of arteriolarwall, or a combination of these signs	Modest association with risk of clinical Stroke, subclinical stroke,coronary heart disease, and death

Moderate retinopathy	Hemorrhage (blot, dot, or flame shaped), microaneurysm cotton-wool spot, hard exudates or a combination of these signs	Strong association with risk of clinical stroke, subclinical stroke, cognitive decline, and cardiovascular mortality

Severe retinopathy	Moderate retinopathy plus optic disc swelling ^#^	Strong association with mortality

*A modest association is defined as an odds ratio of greater than 1 but less than 2. A strong association is defined as anodds ratio of 2 or greater.

^#^Anterior ischemic optic neuropathy, characterized by unilateral swelling of the optic disk, visual loss, and sectorial visual field loss, should be ruled out.

**Table 5:** Initial workup for hypertension.

Complete blood count
Basic metabolic panel including magnesium and phosphate
Serum uric acid
Fasting lipid profile
Fasting blood glucose
Urine analysis/culture
Urine electrolytes, creatinine, protein
Chest X-ray
EKG and echocardiogram
Renal ultrasound with doppler

**Table 6:** Further workup if needed depending upon the etiology.

TSH, Free T4. Free T3
Serum cortisol
Serum aldosterone
Serum renin levels
HbA1C
24 hr urinary catecholamine and metanephrine levels
(Pheochromocytoma)
Serum parathyroid hormone levels
Urine and serum toxicology screen
Urine pregnancy test
CT/MRI scan
DMSA/DTPA scan (renal scars)
MIBG scan (pheochromocytomas)
ANA/ESR/CRP/anti-dsDNA/anti-smith/rheumatoid
factor/pANCA/cANCA

**Table 7:** Commonly used medications for hypertensive crisis.

Medication	Dose and Route	Mechanism of action	Duration of action	Adverse effects	Contraindications and precautions
Sodium nitroprusside	0.5–10 *μ*g/kg/min I.V	Acts by releasing nitric oxide	1-2 minutes	hypotension, palpitations, headache, nausea, vomiting, raised intracranial pressure, thiocyanate and cyanide toxicity, thyroid suppression	Intracranial hypertension

Nicardipine	1–3 *μ*g/kg/min IV	Calcium Channel Blocker	15–30 minutes; may last for up to 3-4 hrs	flushing, hypotension, palpitations, angina, syncope, peripheral edema, headache, vomiting	requires large fluid volume

Esmolol	125–500 *μ*g/kg/min intravenously	Beta-blocker	10–20 minutes	bradycardia, hypotension, bronchoconstriction, skin necrosis after extravasation, Raynaud's phenomenon	Asthma, congestive cardiac failure, cocaine toxicity

Labetalol	0.25–3 mg/kg/hr intravenously	Combined alpha and beta blocker	Up to 4 hrs	bradycardia, hypotension, atrioventricular conduction disturbances, headache, bronchospasm, nasal congestion	

Hydralazine	0.1–0.6 mg/kg/dose every 4–6 hrs intravenously	Direct vasodilatation of arterioles	1–4 hrs	palpitations, flushing, tachycardia, fever, rash, headache, arthralgia, SLE-like syndrome, positive ANA, peripheral neuropathy	

Fenoldopam	0.8–1.2 *μ*g/kg/min intravenously	Dopamine D1 receptor agonist	1 hr	tachycardia, hypotension, flushing, headache, hypokalemia, nasal congestion	

Phentolamine	0.05–0.1 mg/kg/dose Intravenously (maximum of 5 mg per dose)	Alpha-adrenergic blocker	15–30 minutes	tachycardia, palpitations, hypotension, flushing, headache, nasal congestion, exacerbation of peptic ulcer	

Enalaprilat	5–10 mcg/kg/dose every 8–24 hrs intravenously	Angiotensin-converting enzyme inhibitor	4–6 hrs	hypotension, hyperkalemia, oliguria, rash, angioedema, agranulocytosis, neutropenia, cough, fatal hepatic necrosis (rare)	patients with supra-renal aortic stenosis and bilateral renal stenosis; most valuable in neonatal hypertension

Nifedipine	0.1–0.25 mg/kg/dose every 4–6 hrs (maximum 10 mg/dose) oral	Calcium channel blocker	4–8 hrs	Flushing, hypotension, tachycardia, palpitations, syncope, peripheral edema, headache, thrombocytopenia, rash, urticaria, elevated liver enzymes	

Clonidine	0.05–0.1 mg/dose orally	Central alpha-agonist	6–8 hrs	bradycardia, hypotension, rebound hypertension with abrupt withdrawal, sedation, dry mouth,	Avoid sudden discontinuation

Minoxidil	0.1-0.2 mg/kg/day (maximum 5 mg/day) orally	Hyperpolarization of K^+^channels resulting in smooth muscle relaxation	Up to 24 hrs	tachycardia, fluid retention, rash, headache, weight gain, pulmonary edema, Stevens-Johnson syndrome, photosensitivity	Pericardial effusion

Losartan	dose for less than 6 years is not established; for children >6 years 0.7 mg/kg once daily (maximum dose 100 mg/day) orally	Angiotensin II receptor blocker	24 hrs	hypotension, chest pain, hyperkalemia, elevation in BUN/Creatinine, headache, fever, syncope, diarrhea, flu-like illness	Patient with suprarenal aortic stenosis and bilateral renal stenosis.

Clevidipine	0.5–3.5 mcg/kg/min intravenously	L-type calcium Channel blocker	up to 15 minutes	Headache, nausea, vomiting, hypotension	Patients with lipid disorders and egg and soy protein allergies

## Abbreviations

CRP: C-reactive protein
IL: Interleukin
IF: Interferon
TNF: Tumor necrotic factor
ICAM: Intercellular adhesion molecule
VCAM: Vascular cell adhesion molecule
GFR: Glomerular filtration rate.
